# Rare Genetic Variants Associated With Myocardial Fibrosis: Multi-Ethnic Study of Atherosclerosis

**DOI:** 10.3389/fcvm.2022.804788

**Published:** 2022-02-21

**Authors:** Mahsima Shabani, Diptavo Dutta, Bharath Ambale-Venkatesh, Wendy S. Post, Kent D. Taylor, Stephen S. Rich, Colin O. Wu, Naveen L. Pereira, Sanjiv J. Shah, Nilanjan Chatterjee, Jerome I. Rotter, Dan E. Arking, Joao A. C. Lima

**Affiliations:** ^1^Division of Cardiology, Department of Medicine, School of Medicine, Johns Hopkins University, Baltimore, MD, United States; ^2^Department of Biostatistics, Johns Hopkins Bloomberg School of Public Health, Baltimore, MD, United States; ^3^Department of Radiology, Johns Hopkins University, Baltimore, MD, United States; ^4^Department of Pediatrics, The Institute for Translational Genomics and Population Sciences, The Lundquist Institute for Biomedical Innovation at Harbor-University of California, Los Angeles (UCLA) Medical Center, Torrance, CA, United States; ^5^Center for Public Health Genomics, University of Virginia, Charlottesville, VA, United States; ^6^Office of Biostatistics Research, National Heart, Lung, and Blood Institute, National Institutes of Health, Bethesda, MD, United States; ^7^Department of Cardiovascular Medicine, Mayo Clinic, Rochester, MN, United States; ^8^Division of Cardiology, Feinberg Cardiovascular Research Institute, Northwestern University Feinberg School of Medicine, Chicago, IL, United States; ^9^Department of Genetic Medicine, McKusick-Nathans Institute, Johns Hopkins University School of Medicine, Baltimore, MD, United States

**Keywords:** cardiomyopathy, genetics, fibrosis, magnetic resonance imaging, T1, interstitial, rare

## Abstract

**Background:**

Rare pathogenic variants in cardiomyopathy (CM) genes can predispose to cardiac remodeling or fibrosis. We studied the carrier status for such variants in adults without clinical cardiovascular disease (CVD) in whom cardiac MRI (CMR)-derived measures of myocardial fibrosis were obtained in the Multi-Ethnic Study of Atherosclerosis (MESA).

**Objectives:**

To identify CM-associated pathogenic variants and assess their relative prevalence in participants with extensive myocardial fibrosis by CMR.

**Methods:**

MESA whole-genome sequencing data was evaluated to capture variants in CM-associated genes (*n* = 82). Coding variants with a frequency of <0.1% in gnomAD and 1,000 Genomes Project databases and damaging/deleterious effects based on *in-silico* scoring tools were assessed by ClinVar database and ACMG curation guidelines for evidence of pathogenicity. Cases were participants with high myocardial fibrosis defined as highest quartile of extracellular volume (ECV) or native T1 time in T1-mapping CMR and controls were the remainder of participants.

**Results:**

A total of 1,135 MESA participants had available genetic data and phenotypic measures and were free of clinical CVD at the time of CMR. We identified 6,349 rare variants in CM-associated genes in the overall MESA population, of which six pathogenic/likely pathogenic (P/LP) variants were present in the phenotyped subpopulation. The genes harboring P/LP variants in the case group were *MYH7, CRYAB*, and *SCN5A*. The prevalence of P/LP rare variants in cases was higher than controls (5 in 420 [1.1%] vs. 1 in 715 [0.1%], *p* = 0.03). We identified two *MYBPC3* Variants of Unknown Significance (VUS)s with borderline pathogenicity in the case group. The left ventricle (LV) volume, mass, ejection fraction (EF), and longitudinal and circumferential strain in participants with the variants were not different compared to the overall cohort.

**Conclusions:**

We observed a higher prevalence of rare potentially pathogenic CM associated genetic variants in participants with significant myocardial fibrosis quantified in CMR as compared to controls without significant fibrosis. No cardiac structural or functional differences were found between participants with or without P/LP variants.

## Introduction

Myocardial fibrosis is the accumulation of extracellular matrix in the myocardial tissue due to the negligible regenerative capacity of the myocardium (1). It contributes to the pathology of several heart diseases, including dilated and hypertrophic cardiomyopathies (CM) (1). The profibrotic state is an initial step in the process of CM development followed by fibrosis visible in cardiac MRI and evident morphologic changes related to CM (2).

Although, the current gold-standard for detection and quantification of myocardial fibrosis is endomyocardial biopsy, non-invasive imaging techniques such as cardiac MRI (CMR) can provide several indirect markers of myocardial fibrosis (3, 4). Harmonic Phase quantification of tagged CMR evaluates ventricular strain that can identify ventricular dysfunction due to fibrosis-mediated myocardial stiffening (5). Late-Gadolinium Enhancement (LGE) is widely used to illustrate myocardial scars and the replacement fibrosis in the myocardium, reflected by the accumulation of contrast in myocardial tissue (4). T1 mapping is a more recent technique with higher sensitivity thresholds for myocardial fibrosis identification, using direct measurement of extracellular volume (ECV) fraction of myocardial tissue (4). This method has high specificity in detection of myocardial fibrosis and can be easily measured even in those with normal cardiac function (6). Reports show that longer native T1 values depict higher extent of myocardial fibrosis with a 98% diagnostic accuracy in patients with cardiomyopathy (7). ECV, calculated by hematocrit and pre- and post-contrast T1 values in blood and myocardium, is an index of remodeling in interstitial and extracellular spaces and is a better indicator of collagen volume fraction than post-contrast T1 value (8).

The known genetic background of cardiomyopathies includes variants/mutations in genes related to cell contractility, sarcomere proteins, calcium homeostasis, cytoskeleton, and metabolic pathways, which predispose to changes in ventricular structure, function, and remodeling (9–11). Myocardial fibrosis occurs in CM as well as other myocardial pathologies, contributing to structural and functional changes. Previous genome-wide association studies (GWAS) identified tens of variants associated with CMR-based cardiac structure and function, many of which were located in proximity to Mendelian CM genes including *TTN, CDKN1A, BAG3, SH2B3, MYH6*, and *MYH7* (12, 13). However, whether rare pathogenic/likely pathogenic (P/LP) variants in CM genes are associated with myocardial fibrosis, a predecessor of evident morphologic changes in CM, is unknown. Early identification of pathogenic variants in CM-related genes not only would call for a stricter surveillance in CM prevention and progression in proband and relatives (14), but also identifies the target population for the novel yet promising treatment methods (15, 16).

In this study, we aimed to determine if rare P/LP variants (minor allele frequency <0.1%) in CM genes are enriched in participants with high myocardial fibrosis levels. For this analysis, using whole genome sequencing (WGS) data, a custom list of variants in a cardiomyopathy-related gene panel were called using a bioinformatics pipeline designed based on frequency, location of variant, and its predicted deleterious effect. Cases with a high level of myocardial fibrosis were selected and the distribution of rare pathogenic variants were compared between cases and the remainder of cohort. Finally, other CMR-related phenotypic measures were evaluated in the carriers of these variants.

## Methods

### Study Population and Case Definition

The Multi-Ethnic Study of Atherosclerosis (MESA) was initiated in 2000 with a baseline sample of over 6,814 individuals aged 45–84 years, out of whom 4,632 had whole genome sequencing through participation in the TOPMed consortium. MESA includes 38% White, 28% African American, 23% Hispanic, and 11% Chinese American participants recruited from six US field centers (17). CMR late gadolinium enhancement and T1 mapping studies were performed during the 5th MESA exam (2010–11). A total of 1,345 people underwent contrast-enhanced CMR and T1 mapping. The tagging MRI protocol was applied to 3,100 participants with cardiac MRI in exam 5 and global circumferential strain (GCS) was calculated. Since this analysis is an individual-based analysis, no missing data were imputed and participants lacking outcome or covariate data were excluded.

### Myocardial Fibrosis Measurement and Case Selection

The MRI protocol for the assessment of myocardial fibrosis was previously described (18). Native T1 and ECV derived from T1-mapping MRI were used as direct surrogates of interstitial myocardial fibrosis in this study. T1 mapping studies include estimation of T1 times at pre-contrast (native) phase and 12- and 25-min post gadolinium contrast injection. ECV fraction and partition coefficient were calculated. A single breath-hold ECG-synchronized Modified Look-Locker Inversion recovery (MOLLI) approach was used to assess T1 times. The LGE method identified hyperenhancement areas evident in images recorded 15 min after a bolus of gadolinium injection. Hyper-enhanced areas are reported as the presence of focal scars (binomial variable).

Participants with a history of myocardial infarction (MI) or heart failure (HF) were excluded to filter out participants with a potential replacement fibrosis. Cases were individuals with high myocardial fibrosis defined as the highest quartile of ECV in T1 mapping or highest quartile of native T1 in the total population with available CMR measurements. Further information on the CMR or echocardiography techniques and analyses is available in the Supplementary Material. The speckle-tracking echocardiography was performed at MESA exam 6 by the Northwestern University Echocardiography Core Lab (NUECL, Chicago, Illinois) (19). The average of the myocardial strain measured in apical 4-, 3-, and 2-chamber views was reported as the LV global longitudinal strain (GLS).

### Gene Sequencing

Whole genome sequencing was performed in DNA samples obtained at the first MESA exam (2000–2002) as part of the Trans-Omic for Precision Medicine (TOPMed) program of National Heart, Lung, and Blood Institute (NHLBI) (20). There were 4,632 participants who underwent sequencing, of whom 107 (2.3%) were excluded due to withdrawal of consent for genomic analyses, enrollment despite preexisting cardiovascular disease, excess DNA contamination, mean sequencing coverage <30 × , or sample duplicates—resulting a final dataset of 4,525 individuals with sequencing data available for the current analysis. Variants were called using Genome Analysis Toolkit HaplotypeCaller software. The sensitivity of the selected variant quality score recalibration threshold was 99.8% for single-nucleotide polymorphisms as empirically assessed using HapMap controls with known genotypes included in the sequencing call set. In total, 1,164 participants had both the variant calls and the T1 mapping phenotypic measures described above.

### Bioinformatics Pipeline

The variant calls were initially filtered to capture any variant in 82 CM-related genes (Supplementary Table 1). Only non-monomorphic variants in the phenotyped subsample were retained for subsequent analysis. These variants were annotated for a spectrum of genomic, regulatory, and other features using the Variant Effect Predictor (VEP version 86) pipeline developed by Ensembl (21). The worst consequence of the variant was chosen across all transcripts (Transcript database: Ensembl/GENCODE v26). The selection pipeline was designed to further narrow down the list of annotated variants based on following criteria: (1) Located in coding and canonical splice site donor and acceptor sites; (2) Frequency of <0.1% based on gnomAD (v2.1.1) and 1,000 genomes; (3) Non-synonymous; (4) Deleterious (-or no prediction score) predictive effect using PolyPhen and CADD, or damaging effect (-or no prediction score) using SIFT and FATHMM scoring tools; (5) Non-benign evidence in the ClinVar database (Supplementary Table 1). Resultant variants were assessed by the ACMG/Association of Molecular Pathology (AMP) benign/pathogenic variant classification criteria blinded to the case/control status or any other phenotype information, and those with P/LP interpretation were selected (22). The interpretation platforms of InterVar and Varsome were used to decrease reader-based biases (23, 24). VUSs (Variant of Uncertain Significance) with borderline pathogenicity (likely pathogenic after adjustment of ACMG interpretation based on potentially supporting phenotype) were also reported (VUS+).

### Statistical Analysis

The number of rare variants was compared between the cases and the remainder of the population. The mean values of ECV, GLS, GCS, and left ventricle (LV) anatomical measures [including LV mass index, LV end-diastolic volume (EDV) index, LV end-systolic volume (ESV) index, LV mass to volume ratio (MVR), and ejection fraction (EF)], and prevalence of positive LGE was compared between cases harboring the variants vs. cases who did not and the controls. The related quartile of above-mentioned ventricular features was reported for cases with a P/LP variant and their estimated measures in cases were illustrated relative to the distribution in the entire cohort.

Significance for hypotheses testing was set at <0.05 level. All analyses were completed using R statistical programming software, version 3.6.2. Based on our initial calculation, a sample size of 420 cases would provide a power of >80% to detect a 1.5% difference in prevalence of variants in the cases vs. controls with a significance level of *P* < 0.05.

## Results

**Figure 1:**
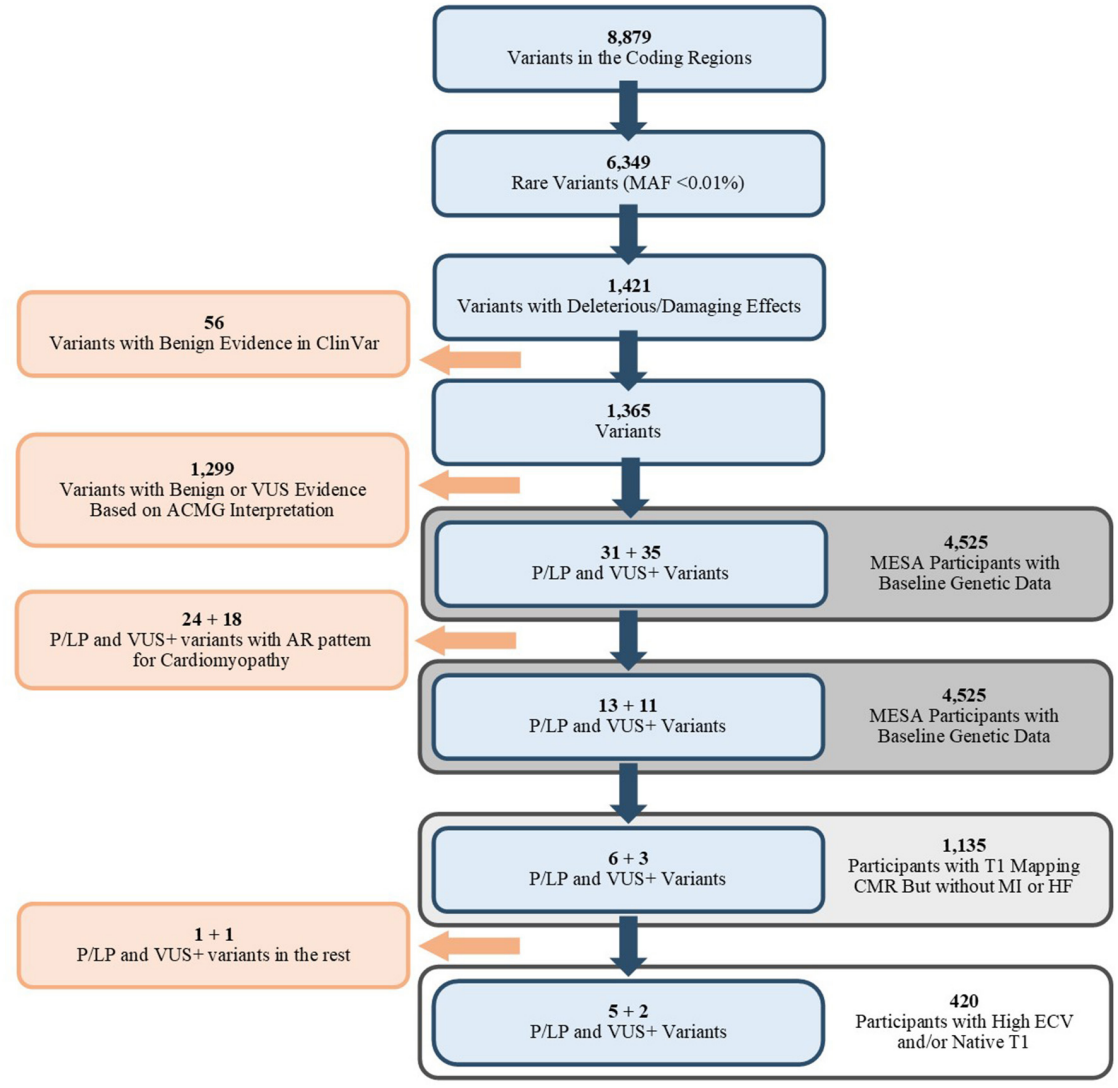
Flowchart of selected variants (Blue) and the associated population. MAF, Mean Allele Frequency; ACMG, American College of Medical Genetics and Genomics; P/LP, Pathogenic/Likely Pathogenic; VUS, Variant of Unknown Significance; VUS+, Variants that were VUS without adjustment of ACMG/AMP criteria; AR, Autosomal Recessive; CMR, Cardiac MRI; MI, Myocardial Infarction; HF, Heart Failure; ECV, Extracellular Volume.

A total of 1,164 MESA participants had available genetic data and the T1 mapping measurements available. Participants who had a history of MI or HF before the time of CMR acquisition (*n* = 29) were excluded from the analysis (Figure 1). There was a total of 420 participants (37%) who had either an ECV level in the fourth quartile only (ECV > 28.8, *n* = 139), a native T1 time in the fourth quartile only (native T1 > 1,006 ms, *n* = 139), or both (*n* = 142). Median age at the time of CMR exam was 69 years in cases with 63% females and 67 years in controls (39% female). There were 55 and 53% of the cases and controls who were white (Table 1).

**Table 1:** Variable distribution among cases vs. controls.

**Variable**	**Cases (*n* = 420)**	**Controls (*n* = 715)**	***P*-value**
Age at MRI	68.6 (9.1)	67.0 (8.4)	**0.002**
**Sex**
Female	265 (63.1%)	277 (38.7%)	**<0.001**
Male	155 (36.9%)	438 (61.3%)	
**Race**
White	232 (55.2%)	379 (53.0%)	0.11
African American	105 (25.0%)	151 (21.1%)	
Chinese American	43 (10.2%)	71 (9.9%)	
Hispanic	40 (9.5%)	114 (15.9%)	
BMI (kg/m^2^)	28.1 (5.7)	28.6 (4.8)	0.12
SBP (mmHg) at CMR	121.8 (19.6)	121.2 (18.4)	0.58
DBP (mmHg) at CMR	67.0 (10.0)	69.3 (9.3)	**<0.001**
Diabetes mellitus	65 (15.7%)	113 (15.9%)	0.80
ECV	29.4 (2.5)	25.6 (1.8)	**<0.001**
Native T1	1,014 (37.1)	957.2 (30.7)	**<0.001**

*BMI, Body mass index; SBP, Systolic blood pressure; DBP, Diastolic blood pressure; ECV, Extracellular volume. Values marked in bold show significant associations*.

Nine P/LP variants or VUS+ were identified among the 1,135 participants (Figure 1). The details of ACMG/AMP interpretation of variants are available in Supplementary Table 2. After unblinding of the case status, 7 variants (5 P/LP, 2 VUS+) in 4 genes (*MYH7, CRYAB, MYBPC3*, and *SCN5A)* were observed in 7 cases, and 2 variants (1 P/LP, 1 VUS+) in 2 distinct genes (*MYL2, TNNT2)* were observed in the remaining 715 participants (Table 2). Among the 7 participants in the case group, two had non-cardiovascular death and one developed HF during follow-up.

**Table 2:** Cases/Controls with pathogenic/likely pathogenic variants and VUS+ in cardiomyopathy genes with AD pattern of inheritance.

	**Age**	**Sex, race**	**Phenotype criteria**	**Gene**	**Type**	**Variant**	**Transcript**	**ClinVar interpretation**	**ACMG interpretation**	**gnomAD AF in ethnic group**	**Associated disease**
**Cases**	
C1	50–60	F, African	High ECV	*SCN5A*	Missense	3:38613773G>A	p.Arg225Trp	P/LP	Pathogenic	1.28e−4	DCM, LVNC
C2	70–80	F, African	High Native T1	*CRYAB*	Missense	11:111908822G>A	p.Arg157His	VUS	Likely pathogenic	6.15e−5	DCM, LVNC
			High ECV								
C3	50–60	M, Chinese	High Native T1	*CRYAB*	Start codon loss	11:111911722G>A	p.Met1Ile	Conflicting	Likely pathogenic	9.84e−4	DCM, LVNC
C4	80–90	F, White	High Native T1	*MYH7*	Missense	14:23424839G>A	p.Arg870His	Pathogenic	Likely pathogenic	1.55e−5	HCM, DCM, LVNC
			High ECV								
C5	60–70	M, African	High Native T1	*MYH7*	Missense	14:23429037C>T	p.Arg442His	Conflicting	Likely pathogenic	6.15e−5	HCM, DCM, LVNC
C6	50–60	M, Hispanic	High ECV	*MYBPC3*	Missense	11:47337543G>A	p.Arg817Gln	Conflicting	VUS+	2.83e−5	HCM, DCM, LVNC
C7	70–80	M, White	High Native T1	*MYBPC3*	Missense	11:47337792G>A	p.Val771Met	Conflicting	VUS+	1.76e−5	HCM, DCM, LVNC
			High ECV								
**Controls**	
N1	50–60	M, Hispanic	–	*MYL2*	Missense	12:110914290C>T	p.Gly57Glu	VUS	Likely pathogenic	2.89e−5	HCM
N2	60–70	M, White	–	*TNNT2*	Missense	1:201365261G>A	p.Ala114Val	Conflicting	VUS+	1.76e−5	HCM, DCM, RCM, LVNC

*VUS+: Variants that were VUS without adjustment of ACMG/AMP criteria.

*ECV, Extracellular volume; DCM, Dilated cardiomyopathy; LVNC, Left ventricular non-compaction; HCM, Hypertrophic cardiomyopathy; RCM, Restrictive cardiomyopathy*.

The prevalence of any P/LP variant or VUS+ in the CM genes was higher in the case group (1.7% [7/420]) vs. the controls (0.3% [2/715], *p* = 0.01). The prevalence of any P/LP variants was higher in the case group (1.2% [5/420]) vs. the controls (0.1% [1/715], *p* = 0.03).

**Figure 2:**
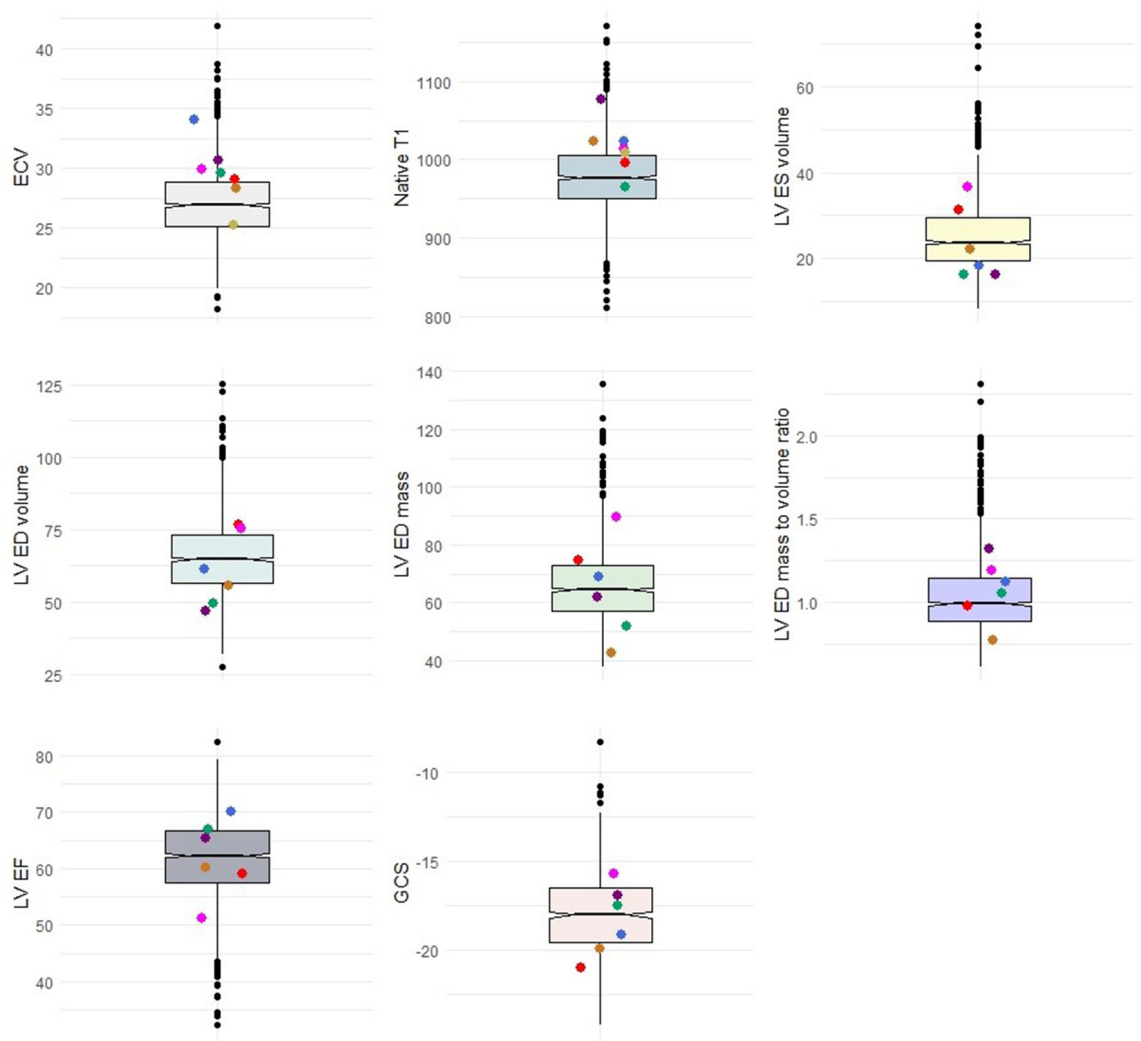
Distribution of cases with P/LP variants or VUS+ (colored dots) in comparison with the total population. Colored dots represent cases. Box plots represent the range of data within the 1st to 3rd quartile of variables with relation to the median in notches. ECV, Extracellular volume; LV, Left ventricle; ES, End-systolic; ED, End-diastolic; EF, Ejection fraction; GCS, Global circumferential strain.

One case with a P/LP variant in *MYH7* had evidence of scar in the LGE analysis (Table 3). All of the cases had an EF of higher than 45% (min: 51.3%). The values of indexed LV mass, LV EDV, LV ESV, and LV MVR were heterogeneous in the carriers of CM variants (Figure 2). However, there were no significant difference in LV end-diastolic mass to volume ratio (1.1 vs. 1.0, *p* = 0.54), LV EF (62.1 vs. 62.0, *p* = 0.97), and other anatomical LV features between cases with CM variants and cases with no CM variants (Table 4). Likewise, there was no difference in anatomical LV features between cases with pathogenic variants and controls (Table 4). Furthermore, there was no difference in functional LV measures, including GCS and GLS, between cases with pathogenic variants and either cases without pathogenic variants (GCS: 18.3 vs. 18.3, *p* = 0.96; GLS: 19.0 vs. 20.1, *p* = 0.61) or controls (GCS: 18.3 vs. 17.9, *p* = 0.57, GLS: 19.0 vs. 19.7, *p* = 0.74). Figure 2 illustrates the distribution of cardiac structural and functional variables in cases with P/LP or VUS+ variants compared to the distribution of each variable in the entire cohort.

**Table 3:** Individual-based description of global circumferential strain and LV anatomical features in cases with P/LP/VUS+ variants.

**#Case**	**Age category**	**Phenotype criteria**	**Gene**	**Variant**	**GCS**	**LV ESV**	**LV EDV**	**LV mass**	**LV ED-MVR**	**LV EF**	**LGE scar**
C1	50–60, F	High ECV	*SCN5A*	3:38613773C>T	Q3	Q1	Q1	Q1	Q3	>45%	No
C2	70–80, F	High Native T1	*CRYAB*	11:111908822G>A	Q1	Q3	Q2	Q1	Q1	>45%	No
		High ECV									
C3	50–60, M	High Native T1	*CRYAB*	11:111911722G>A	Q2	Q2	Q3	Q3	Q3	>45%	No
C4	80–90, F	High Native T1	*MYH7*	14:23424839G>A	Q3	Q1	Q1	Q2	Q4	>45%	No
		High ECV									
C5	60–70, M	High Native T1	*MYH7*	14:23429037G>A	Q4	Q4	Q4	Q4	Q4	>45%	Yes
C6	50–60, M	High ECV	*MYBPC3*	11:47337543G>A	Q1	Q4	Q4	Q4	Q2	>45%	No
C7	70–80, M	High Native T1	*MYBPC3*	11:47337792G>A	NA	NA	NA	NA	NA	NA	No
		High ECV									

*GCS, Global circumferential strain; LV, Left ventricle; ESV, End-systolic volume; EDV, End-diastolic volume; ED-MVR, End-diastolic mass-to-volume ratio; EF, Ejection fraction; LGE, Late gadolinium enhancement; ECV, Extracellular volume*.

**Table 4:** Comparison of LV anatomic features between cases with CM variants and without variants, as well as cases with CM variants and the controls.

**Variable**	**Cases with variant (*n* = 7)**	**Cases without variants (*n* = 413)**	***P*-value**	**Controls (*n* = 715)**	***P-*value**
ECV (%)	29.5 (2.6)	29.5 (2.6)	0.99	25.5 (1.9)	**0.007**
Native T1 (ms)	1,015.1 (33.7)	1,013.2 (39.1)	0.88	955.9 (31.0)	**0.003**
LV EDM (g/mm^2^)	65.0 (13.1)	64.3 (14.3)	0.91	66.4 (12.0)	0.84
LV EDV (mL/mm^2^)	60.9 (12.7)	65.3 (13.7)	0.44	65.2 (13.1)	0.45
LV ESV (mL/mm^2^)	23.5 (8.6)	25.0 (8.3)	0.67	25.1 (7.5)	0.68
LV ED-MVR (g/mL)	1.1 (0.2)	1.0 (0.2)	0.54	1.0 (0.2)	0.74
LV EF (%)	62.1 (6.7)	62.0 (7.6)	0.97	61.8 (6.6)	0.92
GCS by CMR (%)	18.3 (2.0)	18.3 (2.4)	0.96	17.9 (2.2)	0.57
GLS by ST-echo (%)	19.0 (2.1)	20.1 (2.9)	0.61	19.7 (2.7)	0.74

*ECV, Extracellular volume; LV, Left ventricle; EDM, End-diastolic mass; EDV, End-diastolic volume; ESV, End-systolic volume; ED-MVR, End-diastolic mass to volume ratio; EF, Ejection fraction; GCS, Global circumferential strain; GLS, Global longitudinal strain; CMR, Cardiac MRI; ST-echo, Speckle tracking echocardiography. Values marked in bold show significant associations*.

## Discussion

In this study, we observed rare P/LP/VUS+ variants in 4 genes associated with cardiomyopathy (*MYH7, CRYAB, SCN5A, and MYBPC3)* in individuals without a history of MI or HF and with extensive myocardial fibrosis indicated by CMR. The prevalence of P/LP variants in this group was higher (1.1%) than those with lower myocardial fibrosis (0.1%). On average, participants with rare P/LP or VUS+ variants did not have a significantly different LV volume, mass, EF, and longitudinal and circumferential strain at the time of CMR, compared to the overall cohort.

Identification of individuals who carry P/LP variants can be used to screen for genetic risk for cardiomyopathy and consequent cardiac dysfunction or HF in the clinically asymptomatic stage (25, 26). Although the cost and the processing time of genetic sequencing is decreasing, the rare frequency of these variants in the general population, without reliable clinical predictors, makes genetic testing a less ideal screening method. However, the yield of genetic testing may improve in a more focused target population with a higher prevalence of pathogenic variants, in this case, asymptomatic individuals with high myocardial fibrosis in CMR. Identification of pathogenic variants in these participants can subsequently be used for family screening and risk prediction (27, 28).

Previous reports suggest myocardial fibrosis as a marker of risk for future cardiac dysfunction (29, 30). Cardiac autopsy identified myocardial fibrosis as the primary cause of 3.6% of sudden cardiac death in victims of non-ischemic CM (31). Further DNA testing revealed that more than 10% of individuals had a P/LP variant in CM associated genes. None of the individuals harboring the genetic variants had anatomical findings in the heart autopsy suggestive of CM (31). Likewise, although some of the participants with variants in this study had cardiac structural and functional findings suggestive of cardiomyopathy, on average, there was no difference between variant careers and the non-carrier cases or the controls in ventricular volume, mass, EF, and strain values. Therefore, identification of CM variants associated with CMR detected myocardial fibrosis may help stratify asymptomatic patients at risk for sudden death. Whether such patients are more likely to exhibit disease penetrance or develop CM remains to be proven.

Mutations in the genes expressing sarcomere protein components including *MYH7, MYBPC3*, and *MYL2* are found in a significant number of patients with dilated or hypertrophic cardiomyopathy and thus, pathogenic variants in these genes are recommended by ACMG to be reported even as a secondary or incidental finding in genome or exome sequencing (25). We observed P/LP variants in *MYBPC3* and *MYH7* in four participants with high interstitial fibrosis, yet not all had evident myocardial dysfunction. Previous studies have shown that myocardial fibrosis, measured through the level of serum C-terminal pro-peptide of type I procollagen (PICP), was observed in the carriers of pathogenic sarcomere variants in individuals with or without overt cardiomyopathy (2). A comprehensive CMR analysis of 133 HCM patients showed that patients with sarcomere variants had higher ECV and number of segments with LGE compared to patients with mitochondria-related mutations or no mutations (32). Cardiomyopathy caused by variation in sarcomere genes such as *MYBPC3* have been associated with an inflammatory phenotype and subsequent fibrosis (33), and whether the fibrosis detected by CMR in these patients is a result of inflammation remains to be proven.

Finding a well-powered sample size for the identification of rare variants associated with certain diseases with genome-wide significance is a struggle. Guided sampling used in this study where the tails of phenotype distribution are selected aides in increasing the power of these studies (34). However, even though the higher prevalence of P/LP variants in participants with extensive myocardial fibrosis suggests the carrier status for these variants as a potential risk factor, we should note that this study does not have sufficient power to detect a strong association between the observed variants and myocardial fibrosis. In particular, the study population consisted of participants who not only had no CVD at baseline but also survived at least for 10 years (to the 5th MESA exam), making them a healthier population. Thus, we believe that the real prevalence of rare variants with a pathogenic effect in myocardial fibrosis in people with extensive myocardial fibrosis could be even higher. The cases in our study were older and were female-dominant compared to controls, which could also account for the increased fibrosis observed in the case group (30). However, it is well-known that epigenetic factors in the setting of genetic susceptibility contribute to the manifestation of CM (35).

The deep-coverage WGS data provided in the multi-ethnic Trans-Omics for Precision Medicine (TOPMed) genomic resource provides a convenient platform for the detection of rare variants not detectable in similar genome-wide databases (20, 36). On the other hand, MESA offers solid predictors of subclinical CM through its unique CMR-based assessments of cardiac fibrosis, the T1 mapping. To facilitate the comparison, we grouped participants with any LP/P variant together. Nevertheless, the restricted number of carriers of P/LP variants limited this study from evaluating the course of progression of cardiac dysfunction and event rate in these carriers. Moreover, despite using a multi-ethnic cohort, readers should note that the result of this study does not apply to ethnic groups not involved in the MESA study, including South Asians and the non-Chinese Asian population. A limitation of running the variants found in a group of individuals through bioinformatics pipelines is the inability to interpret a few criteria for pathogenicity or benign status of variant. For example, *de novo* status (PS2) or segregation in relatives (BS4, PP1) cannot be determined with no family studies.

Additional longitudinal assessment of carriers of these variants and validation studies in other cardiovascular cohorts may set the stage for the use of myocardial fibrosis with CM gene-panel assessment as a preventive and personalized cardiology tool in people at risk for cardiomyopathy. Early preventative care in these carriers coupled with effective control of comorbidities may attenuate the development of HF (14). Moreover, with the discovery of novel and promising therapeutic approaches, including sarcomere protein inhibitors (15) and base editors (16), carriers of pathogenic variants in CM-related genes can be treated, if not cured.

## Data Availability Statement

Publicly available datasets were analyzed in this study. This data can be found here: MESA website (https://www.mesa-nhlbi.org/).

## Ethics Statement

The studies involving human participants were reviewed and approved by all field centers and core labs of the MESA study including: Columbia University, New York Johns Hopkins University, Baltimore Northwestern University, Chicago UCLA, Los Angeles University of Minnesota, Twin Cities Wake Forest University, Winston Salem University of Washington Coordinating Center University of Vermont Laboratory UCLA Medical Center Research and Education Institute New England Medical Center The Lundquist Institute for Biomedical Innovation at Harbor-UCLA Medical Center University of Virginia Broad Institute of MIT and Harvard National Heart, Lung, and Blood Institute. The patients/participants provided their written informed consent to participate in this study.

## Author Contributions

MS, DD, NC, and DA analyzed the genetic data. MS and DD drafted the manuscript. SS, NP, JR, KT, and SR helped with genetic and phenotype data acquisitions. JR, SR, DA, WP, BA-V, and JL helped with the study design. CW, DA, and JL supervised the biostatistical analysis. JL, DA, CW, WP, JR, and BA-V revised the draft. All authors contributed to the article and approved the submitted version.

## Funding

WGS for the TOPMed program was supported by the National Heart, Lung and Blood Institute (NHLBI). WGS for ‘NHLBI TOPMed: Multi-Ethnic Study of Atherosclerosis (MESA) (phs001416.v1.p1) was performed at the Broad Institute of MIT and Harvard (3U54HG003067-13S1). Centralized read mapping and genotype calling, along with variant quality metrics and filtering were provided by the TOPMed Informatics Research Center (3R01HL-117626-02S1, contract HHSN268201800002I). Phenotype harmonization, data management, sample-identity QC, and general study coordination, were provided by the TOPMed Data Coordinating Center (3R01HL-120393; U01HL-120393; contract HHSN268180001I). The MESA project is conducted and supported by the National Heart, Lung, and Blood Institute (NHLBI) in collaboration with MESA investigators. Support for MESA is provided by contracts 75N92020D00001, HHSN268201500003I, N01-HC-95159, 75N92020D00005, N01-HC-95160, 75N92020D00002, N01-HC-95161, 75N92020D00003, N01-HC-95162, 75N92020D00006, N01-HC-95163, 75N92020D00004, N01-HC-95164, 75N92020D00007, N01-HC-95165, N01-HC-95166, N01-HC-95167, N01-HC-95168, N01-HC-95169, UL1-TR-000040, UL1-TR-001079, and UL1-TR-001420. Also supported in part by the National Center for Advancing Translational Sciences, CTSI grant UL1TR001881, and the National Institute of Diabetes and Digestive and Kidney Disease Diabetes Research Center (DRC) grant DK063491 to the Southern California Diabetes Endocrinology Research Center.

## Conflict of Interest

The authors declare that the research was conducted in the absence of any commercial or financial relationships that could be construed as a potential conflict of interest. The handling editor declared a past co-authorship with one of the authors with the authors WP, KT, SR, JR, and JL.

## Publisher's Note

All claims expressed in this article are solely those of the authors and do not necessarily represent those of their affiliated organizations, or those of the publisher, the editors and the reviewers. Any product that may be evaluated in this article, or claim that may be made by its manufacturer, is not guaranteed or endorsed by the publisher.
