# Supplementary material for: Rare Genetic Variants Associated With Myocardial Fibrosis: Multi-Ethnic Study of Atherosclerosis
Source: Front Cardiovasc Med. 2022 Feb 21;9:804788. doi: 10.3389/fcvm.2022.804788 (PMC8899004; doi:10.3389/fcvm.2022.804788)
Supplement: Supplementary file 1 [file Table_1.docx]

## Supplementary table

Table S1. Number of variants in cardiomyopathy-related genes after each of the initial three steps of variant selection in the pipeline (1. Coding region; 2. Rare frequency (<0.1%); 3. Damaging/Deleterious effect). The last column shows the number of variants after exclusion of Benign variants according to ClinVar.

| **Gene** | **Chromosome** | **Coding Variants** | **Rare Coding Variants** | **Rare Coding Variants with Damaging effects** | **Non-Benign by ClinVar** |
| --- | --- | --- | --- | --- | --- |
| **AGL** | 1 | 141 | 98 | 29 | 28 |
| **CPT2** | 1 | 77 | 52 | 25 | 23 |
| **ACTN2** | 1 | 83 | 69 | 31 | 30 |
| **LMNA** | 1 | 89 | 54 | 15 | 15 |
| **RYR2** | 1 | 252 | 192 | 57 | 57 |
| **TNNT2** | 1 | 54 | 30 | 5 | 5 |
| **PPCS** | 1 | 15 | 7 | 2 | 2 |
| **RIT1** | 1 | 18 | 9 | 3 | 3 |
| **TNNI3K** | 1 | 91 | 66 | 17 | 17 |
| **NRAS** | 1 | 7 | 4 | 0 | 0 |
| **ALMS1** | 2 | 399 | 293 | 67 | 65 |
| **DES** | 2 | 46 | 36 | 13 | 13 |
| **SOS1** | 2 | 67 | 44 | 10 | 9 |
| **TTN** | 2 | 3179 | 2350 | 186 | 178 |
| **PPP1CB** | 2 | 16 | 10 | 0 | 0 |
| **DNAJC19** | 3 | 9 | 8 | 1 | 1 |
| **MRAS** | 3 | 9 | 4 | 0 | 0 |
| **PCCB** | 3 | 90 | 60 | 22 | 21 |
| **MYL3** | 3 | 18 | 13 | 5 | 5 |
| **RAF1** | 3 | 34 | 18 | 3 | 3 |
| **SCN5A** | 3 | 166 | 133 | 54 | 46 |
| **TMEM43** | 3 | 70 | 48 | 17 | 17 |
| **TNNC1** | 3 | 5 | 3 | 0 | 0 |
| **RASA1** | 5 | 56 | 44 | 3 | 2 |
| **SDHA** | 5 | 72 | 46 | 16 | 15 |
| **SLC22A5** | 5 | 50 | 41 | 21 | 21 |
| **SGCD** | 5 | 42 | 27 | 6 | 6 |
| **PLN** | 6 | 2 | 1 | 0 | 0 |
| **EYA4** | 6 | 45 | 26 | 8 | 8 |
| **MTO1** | 6 | 100 | 58 | 24 | 22 |
| **DSP** | 6 | 200 | 154 | 56 | 56 |
| **FLNC** | 7 | 240 | 198 | 86 | 82 |
| **TBX20** | 7 | 22 | 15 | 5 | 5 |
| **PRKAG2** | 7 | 66 | 46 | 14 | 14 |
| **BRAF** | 7 | 37 | 15 | 0 | 0 |
| **TMEM70** | 8 | 11 | 8 | 3 | 3 |
| **DOLK** | 9 | 42 | 25 | 7 | 7 |
| **FKTN** | 9 | 50 | 21 | 10 | 10 |
| **BAG3** | 10 | 65 | 51 | 13 | 13 |
| **RBM20** | 10 | 123 | 94 | 32 | 31 |
| **SHOC2** | 10 | 16 | 14 | 1 | 1 |
| **VCL** | 10 | 76 | 57 | 19 | 19 |
| **CBL** | 11 | 68 | 45 | 19 | 16 |
| **CRYAB** | 11 | 30 | 18 | 7 | 7 |
| **CSRP3** | 11 | 26 | 18 | 11 | 11 |
| **HRAS** | 11 | 19 | 13 | 0 | 0 |
| **MYBPC3** | 11 | 144 | 118 | 52 | 50 |
| **CACNA1C** | 12 | 95 | 73 | 14 | 14 |
| **ABCC9** | 12 | 80 | 52 | 8 | 8 |
| **KRAS** | 12 | 12 | 9 | 2 | 2 |
| **MYL2** | 12 | 25 | 16 | 6 | 6 |
| **PKP2** | 12 | 110 | 79 | 15 | 13 |
| **PTPN11** | 12 | 21 | 19 | 3 | 3 |
| **PCCA** | 13 | 70 | 42 | 18 | 18 |
| **MYH7** | 14 | 88 | 68 | 25 | 22 |
| **SOS2** | 14 | 86 | 62 | 13 | 12 |
| **ALPK3** | 15 | 187 | 141 | 50 | 50 |
| **HCN4** | 15 | 83 | 58 | 7 | 5 |
| **ACTC1** | 15 | 7 | 4 | 1 | 1 |
| **SPRED1** | 15 | 30 | 23 | 4 | 3 |
| **TPM1** | 15 | 52 | 34 | 2 | 2 |
| **MAP2K1** | 15 | 15 | 9 | 2 | 2 |
| **MYLK3** | 16 | 77 | 57 | 10 | 10 |
| **ACADVL** | 17 | 96 | 66 | 26 | 26 |
| **ELAC2** | 17 | 89 | 69 | 26 | 25 |
| **GAA** | 17 | 115 | 91 | 33 | 31 |
| **NF1** | 17 | 117 | 74 | 14 | 14 |
| **JUP** | 17 | 83 | 63 | 26 | 26 |
| **TCAP** | 17 | 19 | 17 | 6 | 5 |
| **DSC2** | 18 | 78 | 60 | 13 | 12 |
| **DSG2** | 18 | 111 | 77 | 22 | 21 |
| **TTR** | 18 | 16 | 10 | 2 | 2 |
| **FKRP** | 19 | 62 | 34 | 15 | 15 |
| **MAP2K2** | 19 | 40 | 22 | 7 | 5 |
| **TNNI3** | 19 | 24 | 16 | 2 | 2 |
| **LZTR1** | 22 | 85 | 62 | 19 | 19 |
| **LAMP2** | X | 27 | 18 | 7 | 6 |
| **TAZ** | X | 24 | 12 | 4 | 4 |
| **FHL1** | X | 20 | 7 | 1 | 1 |
| **GLA** | X | 8 | 5 | 1 | 1 |
| **DMD** | X | 286 | 143 | 41 | 41 |
| **EMD** | X | 4 | 3 | 1 | 1 |
| **Total** | - | 8879 | 6349 | 1421 | 1365 |

Table S2. The details of ACMG interpretation of the identified P/LP/VUS+ variants. In this table, the PP4 level of evidence was added to the variants found in the case group only.

| Gene | Type | Variant | Transcript | ClinVar interpretation | ACMG interpretation | gnomAD AF in ethnic group | ACMG Criteria |
| --- | --- | --- | --- | --- | --- | --- | --- |
| *SCN5A* | Missense | 3:38613773G>A | p.Arg225Trp | P/LP | Pathogenic | 1.28e-4 | PP5, PM1, PM2, PM5, PP3, PP4, BP1 |
| *CRYAB* | Missense | 11:111908822G>A | p.Arg157His | VUS | Likely pathogenic | 6.15e-5 | PP3, PP4, PP5, PM1, PM2 |
| *CRYAB* | Start codon loss | 11:111911722G>A | p.Met1Ile | Conflicting | Likely pathogenic | 9.84e-4 | PVS1, PP3, PP4, PP5, BS2 |
| *MYH7* | Missense | 14:23424839G>A | p.Arg870His | Pathogenic | Likely pathogenic | 1.55e-5 | PM1, PM2, PM5, PP2, PP3, PP5, PP4 |
| *MYH7* | Missense | 14:23429037C>T | p.Arg442His | Conflicting | Likely pathogenic | 6.15e-5 | PM1, PM2, PM5, PS3, PP3, PP4 |
| *MYBPC3* | Missense | 11:47337543G>A | p.Arg817Gln | Conflicting | VUS+ | 2.83e-5 | PM1, PM2, PP3, PP4 |
| *MYBPC3* | Missense | 11:47337792G>A | p.Val771Met | Conflicting | VUS+ | 1.76e-5 | PM1, PM2, PP3, PP5, PP4 |
| *MYL2* | Missense | 12:110914290C>T | p.Gly57Glu | VUS | Likely pathogenic | 2.89e-5 | PM1, PM2, PP2, PP3 |
| *TNNT2* | Missense | 1:201365261G>A | p.Ala114Val | Conflicting | VUS+ | 1.76e-5 | PM1, PM2, PP3, BP1 |

*VUS: Variant of unknown significance; P/LP: Pathogenic/Likely pathogenic; PP: Supporting evidence of pathogenicity; PM: Moderate evidence of pathogenicity; BP: Supporting evidence of benign impact; PVS: Very strong evidence of pathogenicity; BS: Strong evidence of benign impact; PS: Strong evidence of pathogenicity*
